# The Integrated Care Team: A primary care based-approach to support older adults with complex health needs

**DOI:** 10.1177/08404704241293051

**Published:** 2024-10-22

**Authors:** George A. Heckman, Sarah Gimbel, Chantelle Mensink, Brittany Kroetsch, Aaron Jones, Anooshah Nasim, Melissa Northwood, Jacobi Elliott, Adam Morrison

**Affiliations:** 1 8430 University of Waterloo, Waterloo, Ontario, Canada.; 2 6221 Western University, London, Ontario, Canada.; 3 New Vision Family Health Team, Kitchener, Ontario, Canada.; 4 3710 McMaster University, Hamilton, Ontario, Canada.; 5 151158 Lawson Research Institute, London, Ontario, Canada.; 6 Provincial Geriatrics Leadership Ontario, Toronto, Ontario, Canada.

## Abstract

Many older adults have complex needs and experience high rates of acute care use and institutionalization. Comprehensive Geriatric Assessment (CGA) is a specialized multidimensional interprofessional intervention to prevent such outcomes, but access to CGA in the community is limited. The Integrated Care Team (ICT) is a proactive case-finding intervention to support older adults with complex needs in primary care. The ICT provides nurse practitioner-led shared-care supported by a pharmacist, family physician, and geriatrician. Patients undergo a CGA, and a person-centred plan of care is implemented. We conducted a mixed-methods evaluation of the ICT. Patients were 81 ± 9.2 years old, 71% were women. Patients had a high burden of dementia and multimorbidity and received 12.8 ± 5.8 prescriptions daily. The ICT improved prescribing and reduced emergency department visits by 49.5% (*P* = 0.0001). Patients, care partners, and referring physicians reported high satisfaction with care. The ICT is currently being expanded to support additional primary care providers.

## Introduction

Consider an 82-year-old man with two recent emergency department visits, one for heart failure and one for a fall. He has a history of hypertension and type 2 diabetes (complicated by moderate renal impairment, painful peripheral neuropathy, and visual impairment). He has mild dementia, causing difficulties with adherence to a complex medicine regimen. He dislikes taking diuretics as this leads to urinary incontinence. He no longer drives, and his care partner (his spouse) is feeling overwhelmed.

Many older adults today face complex health challenges arising from multimorbidity, mental health conditions, frailty, and functional and cognitive decline. Health outcomes are often suboptimal, with high rates of acute care utilization, prolonged hospitalization, and premature institutionalization.^1-3^ Resultant Emergency Department (ED) and hospital overcrowding has severe repercussions across the healthcare system.^4^ In response, healthcare decision makers focus on downstream solutions, such as transitional care units and more long-term care beds, to improve “patient flow.” These measures have limited impact mainly because they do not consider the needs of these older adults.^5,6^

Older adults with complex needs face a fragmented, under-resourced, and hard-to-navigate healthcare system with limited capacity to meet these needs in a timely manner.^7^ Many could benefit from a Comprehensive Geriatric Assessment (CGA), which prevents ED visits, hospitalization, and premature institutionalization, and improves quality of life.^8^ However, community access to CGA is limited by a shortage of specialized geriatric services for which wait times are lengthy, and because most geriatricians work in hospital settings.^9-11^

To better meet the needs of this population, calls have been made for more primary care teams which have the potential to increase patient and provider satisfaction and access, care coordination, and reduce acute care utilization and healthcare costs.^12^ However, despite greater satisfaction with team-based care among patients and family physicians, system impacts are unclear.^13-22^ Evidence suggests that successful teams are those with a person-centred vision, shared decision-making and leadership, optimized clinician scope of practice, a clear division of labour, communication, collaboration, and conflict resolution, and that use electronic medical records to support quality assurance.^14,23-28^ However, the optimal approach to operationalizing primary care teams to support older adults with complex needs remains to be determined.^7,18,19,29,30^

We previously reported how one Ontario Family Health Team successfully implemented a suite of interprofessional chronic disease management programs for hypertension, diabetes, pharmacist-led anticoagulation, memory care, and heart failure.^31^ However, an important minority of patients, particularly those who were older, were receiving fragmented care in multiple programs. In response, the interprofessional Integrated Care Team (ICT) was created to reduce fragmentation and improve efficiency. The goals of the ICT are to promote ageing in the community, optimize quality of life, support self-management of chronic conditions, and avoid ED visits. Older adults with complex needs referred to the ICT undergo a CGA, and a comprehensive care plan is developed to meet these needs. The purpose of this manuscript is to describe this program and its impact on care efficiency, patient outcomes, and ED visits.

## Description of the Integrated Care Team

The ICT was established in 2017 at the New Vision Family Health Team (NVFHT) in Kitchener, Ontario, Canada. The NVFHT is staffed by 15 physicians and over 40 allied health professionals and serves over 26,500 patients, 13% of whom are over the age of 65 years. The ICT is a shared-care program with Nurse Practitioners (NPs), family doctors, a geriatrician, and a clinical pharmacist.

Patients rostered to the NVFHT access the ICT either through family physician referral, identification of multiple ED visits or falls, or case-finding using the interRAI Assessment Urgency Algorithm (AUA).^32-34^ The AUA is a brief questionnaire assessing functional capacity, shortness of breath, self-rated health, mood, continence, and caregiver burden. Scores range from 1 to 6, with higher scores indicating a more urgent need for CGA.

Referred patients and those with AUA scores of 4 to 6 undergo a CGA.^35^ The Canadian Geriatric Society 5M template was initially used but physical distancing measures required by COVID-19 pandemic measures led to the adoption of the interRAI Check-Up Self-Report instrument.^36-39^ The Check-Up is a standardized, valid, and reliable 30-minute instrument shared with and completed electronically by patients with their care partners, or completed over the phone with the support of a team member. Check-Up outputs include risk and severity measures such as the Cognitive Performance Scale version 2 (CPS2), self-report mood scale, basic and Instrumental Activities of Daily Living Hierarchies (ADLH/IADLH), pain, fall risk (defined as having had at least one fall in the 90 days prior to assessment), continence, caregiver burden, frailty-related health instability (Changes in Health, End-stage disease Signs and Symptoms - CHESS scale), risk of ED visits (DIVERT - Detection of Indicators and Vulnerabilities for Emergency Room Trips), and socioeconomic challenges.^40-44^ A medication review is conducted in tandem to support deprescribing and prevent adverse drug reactions.

The initial clinic visit is scheduled for 90 to 120 minutes. The prior completion of the Check-Up allows for more focused use of time to explore patient and care partner needs, goals, and wishes. A collaborative care plan is developed, including referrals to the in-house geriatrician, other community programs and services, and promoting self-care skills, chronic disease management, and advance care planning. Follow-up includes phone, in-person, or home visits, depending on patient needs. The NP becomes the main contact for follow-up of ICT patients, ensuring rapid access for urgent needs. The geriatrician is available for ad hoc case discussions and, if needed, in-person follow-ups. Care integration is facilitated through a shared electronic medical record and case management provided mainly by the NP and clinical pharmacist, who establish formal links with community providers. The NP supports patient transitions to and from EDs and acute care. Most ICT patients are followed indefinitely because of the chronicity of health needs. Those remaining stable through self-care skill acquisition and appropriate community supports are discharged back to their family physician.

## Methods

Our evaluation was carried out in two phases using mixed methods. Phase 1 examined the ability of the ICT to identify older adults with complex needs and its impact on primary care visits. Phase 2 examined the impact of the ICT on prescribing and ED visits, and on the experiences of care partners and providers.

### Phase 1

Charts of patients enrolled during the first 18 months (2017-2018) of ICT operation and followed for at least one year were reviewed. Variables recorded included patient demographics and clinical characteristics including AUA score, the number of major medical diagnoses and medications, and clinical encounters with NVFHT providers.^45,46^

### Phase 2

A cross-sectional chart review of current ICT patients was conducted during October 2021-November 2021. Variables of interest included demographics, diagnoses, and geriatric syndromes. Outcomes of interest included referrals (geriatric medicine, geriatric psychiatry, and community services), medication optimization, and annualized ED visit rates. The within-person difference in ED visits comparing the year before and year after enrolment in the program was analyzed with a Wilcoxon signed-rank test. Analyses were conducted using SAS 9.4.

In March 2022, we interviewed care partners and family physicians of patients receiving care in the ICT. Care partners were contacted by phone by the NP to introduce the study and invite them to speak with the research assistant. Informed consent was obtained by the research assistant. Clinicians were invited by email, encouraging them to contact the research assistant if interested. One-on-one interviews were conducted with care partners and a focus group with providers. Interviews and focus groups were taped and transcribed verbatim. Members of the research team performed conventional content analysis of the transcripts to generate key themes.^47^

## Results

### Phase 1

We identified 44 patients (29 women) who had been actively followed by the ICT for at least one year. Their mean age was 77.5 ± 10.1 years. Patients had on average 7.2 ± 2.4 major comorbidities and were prescribed 10.8 ± 4.6 medications. AUA scores were skewed to higher risks, with 80% over 3, 53% over 4, and a mode of 6.

Patients followed by the ICT had more encounters with NVFHT providers in the year following enrolment compared to the year prior (849 vs. 496). However, half of these were by phone, with in-person visits decreasing from 496 to 425. The number of visits with a family physician decreased from 213 to 98, with more visits taking place with the NP or another provider.

### Phase 2 - Quantitative

Table 1 describes the 76 patients enrolled in the ICT during October 2021-November 2021. Their mean age was 81.1 ± 9.2 years, and 71% were women. Of the 72 ICT patients with an available AUA, 54 (75%) had a score from 3 to 6, with 30 having a score of 6. Patients had a high burden of cardiorespiratory and neuropsychiatric conditions, and 90.8% had three or more chronic conditions. Almost half had a diagnosis of dementia, half a diagnosis of osteoporosis, and almost two-thirds were experiencing urinary incontinence. Polypharmacy was prominent, a particular concern given a high prevalence of renal insufficiency and the associated risk of adverse drug events.

**Table 1.** Clinical characteristics of ICT patients.

Variables	Total	Male	Female
Frequency (percent)	76 (100)	22 (28.9)	54 (71.1)
Age (mean, SD)	81.1 (9.2)	82.6 (8.2)	80.5 (9.6)
Multimorbidity (3+ chronic conditions)	69 (90.8)	21 (27.6)	48 (63.2)
Alzheimer’s dementia or related dementia	36 (47.4)	11 (14.5)	25 (32.9)
Movement disorder	8 (10.5)	4 (5.3)	4 (5.3)
Depression	20 (26.3)	4 (5.3)	16 (21.1)
Coronary artery disease	18 (23.7)	9 (11.8)	9 (11.8)
Hypertension	50 (65.8)	15 (19.7)	35 (46.1)
Diabetes	29 (38.2)	11 (14.5)	18 (23.7)
Atrial fibrillation	14 (18.4)	6 (7.9)	8 (10.5)
Heart failure	13 (17.1)	6 (7.9)	7 (9.2)
Stroke	18 (23.7)	3 (3.9)	15 (19.7)
Chronic obstructive pulmonary disease and/or asthma	20 (26.3)	5 (6.6)	15 (19.7)
Osteoporosis	44 (57.9)	9 (11.8)	35 (46.1)
Urinary incontinence	49 (64.5)	13 (17.1)	36 (47.4)
Chronic renal failure	45 (59.2)	17 (22.4)	28 (36.8)
Medications (mean, SD)	12.8 (5.8)	11.6 (5.1)	13.3 (6.0)

Abbreviations: SD = standard deviation.

Due to a later introduction, Check-Up outputs were available for only 26 of reviewed patients. Frailty-related health instability (CHESS ≥ 3/5) was present in 11.5%, a very high risk for ED visits (DIVERT ≥ 4/6) in 19.2%, a significant burden of depressive symptoms (self-rated mood ≥ 3/9) in 50%, and daily or moderately severe pain in 34.6%. Cognitive function, based on the CPS2, was intact in 19.2%, mildly impaired in 30.8%, and more severely impaired in 50%. Basic activities of daily living were impaired for 19.2% of patients and 42.3% had moderate or greater deficits with instrumental activities of daily living. An elevated fall risk was noted in 34.6% of patients and cardiorespiratory concerns in 38.5%.

Table 2 presents the outcomes of ICT patients. The in-house geriatrician was involved with over 80%, in-person for most, and 7 were referred to geriatric psychiatry services. Over half received a new referral to community services. Medications were optimized for over half of ICT patients, with on average one medication discontinued (primarily non-steroidal anti-inflammatory drugs, proton pump inhibitors, or psychotropics) and one medication optimized (bone health, cardiovascular, and analgesia). Prior to attending the ICT, patients collectively had had 81 ED visits, with 29 patients having had none. While being followed by the ICT, patients collectively had 38 ED visits, with 42 patients having had none in the subsequent year. Annualized ED visit rates fell by 49.5% (*P* = 0.0001) following enrolment in the ICT.

**Table 2.** Outcomes associated with enrolment in the ICT.

Outcomes	Total	Male	Female
Frequency (percent)	76 (100)	22 (28.9)	54 (71.1)
Geriatrician encounter			
Consult	54 (71.1)	16 (21.1)	38 (50.0)
eReview	9 (11.8)	3 (3.9)	6 (7.9)
New community referrals	40 (52.6)	12 (15.8)	28 (36.8)
Medications deprescribed	32 (42.1)	10 (13.2)	22 (28.9)
Mean (SD)	0.8 (1.3)	1.0 (1.6)	0.8 (1.1)
Medications optimized	46 (60.5)	12 (15.8)	34 (44.7)
Mean (SD)	1.1 (1.2)	1.0 (1.3)	1.1 (1.2)
ED visits – 1 year before (mean, SD)	1.3 (1.5)	1.3 (1.6)	1.3 (1.4)
ED visits – 1 year after (mean, SD)	0.7 (0.9)	0.7 (0.9)	0.6 (0.9)

Abbreviations: SD = standard deviation.

### Phase 2 - Qualitative

Interviews were conducted with eight care partners and one focus group with four family physicians. Due to the small sample size and single program site, demographic information is not being reported. Content analysis identified three themes related to benefits of the ICT over usual care: (1) comprehensive person-centred care; (2) interprofessional collaboration and sharing of care; and (3) system navigation and integration (Table 3).

(1) Care partners perceived that the ICT provides better and more person-centred care, prevents ED visits, and allows some patients to remain at home longer than their family physician would otherwise have expected. Respondents identified that both direct clinical interventions and self-care coaching are important mechanisms for better care. Care partners appreciated the efficient and responsive communication with the ICT.
(2) Respondents also commented favourably on interprofessional collaboration and sharing of care. Care partners reflected that interprofessional collaboration within the ICT allows for more thoughtful and timely care provision, particularly in urgent situations. These observations were echoed by family physicians who noted how support from the ICT helps alleviate their own professional burden.
(3) Finally, respondents all noted how the NP role within the ICT fosters greater system integration and navigation. The NP both facilitates referrals to relevant community services and ensures that these are carried out, often avoiding ED visits. The NP ensures more seamless transitions for patients needing access to acute care.

**Table 3.** Findings from the care partner interviews and family physician focus groups.

Theme	Representative quotes
Comprehensive person-centred care	**Care partners**
	• It was excellent care. I think because of his situation, his depression, his denial, and his grief. They tried their best to keep his physical health and pain at a minimum
	• [Care recipient] had a nasty wound on his leg and they [home and community care] could not send anybody for over a month. So [NP] helped me. She asked me to send her photographs, which I did and she arranged for a prescription to be delivered for antibiotic cream and explained what I should do to care for it… she called back then next weekend, “How’s it going and everything there,” she was just a tremendous help
	• I would say that it had helped me to be more alert to changes. And being able to be proactive and getting him looked at based on identifying changes in his behaviour or physical, mental, or whatever. I’m able to spot them a little earlier
	• I really enjoy working with [NP]. She’s been absolutely awesome, communication wise, you know, by phone, by e-mail like the communication has been there… And I asked a lot of questions. Why, why, why, what, what, what, how? So [NP] has been a godsend and being able to communicate quickly with me because waiting creates more anxiety for me
	**Family physicians**
	• I think it’s felt like a lifesaver for me and for some of my patients with a lot of needs and especially their caregivers. I think it’s been wonderful to have kind of a point person who seems to be aware of a lot of different things going on, so it’s been really helpful
	• He lived in the community way longer than I thought he would have managed, and that was what he wanted
Interprofessional collaboration and sharing of care	**Care partners**
	• [NP] has been our nurse practitioner for the last two years so she would constantly be in touch with us and with [geriatrician] and pharmacist to make sure … for adjusting his medication that was great. Something that we would probably have to take my dad to hospital and they will try to facilitate this medication adjustment. She was just there constantly and so we were able to adjust my dad’s medication
	• The doctor came here a couple of months ago and my husband has Parkinson’s and he wanted to increase his medication. But we had talked to [NP] about this and she checked with the pharmacist and they said no. He was on the maximum and she said she would check that out elsewhere and that they did have a geriatrician that helped him out. So, Dr actually came out here to our home and sat with [patient] for over an hour going over the problems, the timings of when he took the medication. All this stuff and yes, he fully agreed with adding an extra pill a day, so he was a tremendous help
	**Family physicians**
	• I think it helps a little bit with physician burnout
	• But I feel like it’s been a nice balance of [NP] being able to kind of run things by us and update us, and so that we’re kind of still involved. It doesn’t feel as overwhelming as if we had to do it all ourselves
	• I like totally trust that they’ll communicate with me and keep me in the loop about important things
System navigation and integration	**Care partners**
	• [NP] was the go between. She was the liaison between the hospital and us
	• She had been walking around for a month with a fractured pelvis thinking it was sciatica and getting sent back from the emergency room three times. So, it was good because … they said no, go take her now and don’t take no for an answer
	**Family physicians**
	• I have a patient in the program whose daughter called in this morning and was like the patient’s husband is not doing well, caregiver burnout. That sort of thing and they’re on the verge of just taking her to the hospital where it’s [NP], well, let me see what I can do. She made a couple calls and see if you can get any sort of like emergency respite sort of stuff, and it just wouldn’t have been feasible for me in terms of doing any of that
	• I think it’s the making things happen that is the biggest piece. I think we’re all quite competent at providing the medical care, but we’re not great system navigators and it takes a lot of time
	• When our patients do deteriorate and need to go into hospital or are admitted, especially during COVID where family members couldn’t easily kind of communicate, I know that it was great to have [NP] be able to communicate with people at the various emergency rooms or hospitals to help facilitate kind of what is going on

## Discussion

The ICT is a shared-care interprofessional model of care, based in primary care, providing CGA and case management to community-dwelling older adults with complex needs. The ICT improves care efficiency and navigation, provides high quality care, better prescribing, increased access to community services, and reduces ED visits.

The ICT meets several of the quintuple aims, improving patient experience and outcomes, provider experience, and health equity by explicitly targeting and meeting the needs of older adults with complex needs and who would otherwise be unable to access specialized geriatric services in a timely manner.^48^ While our study design precludes an economic evaluation, the reduction in primary care and ED visits suggests potential for cost containment.

The ICT promotes primary care-based system integration and navigation, facilitated by case management provided by the NPs. The NP role in ICT seems well-suited to supporting care transitions to and from acute care. Hospital-based transitional care programs, inherently of limited duration, rarely foster primary care capacity to support older adults with complex needs, which may partly explain the variable success of such programs.^49,50^ The use of the Check-Up brings to primary care a clinical language used in other sectors of healthcare, including home care, community support services, and inpatient mental health, where interRAI instruments are also used, an essential element for system integration.^7,51-53^

The ICT supports health system capacity building at several levels. First, the interprofessional and shared-care approach of the ICT increases primary care capacity to support older adults with complex needs. We have previously shown how such greater capacity to follow and manage patients helps reduce the need for geriatricians to book follow-up appointments and increases geriatrician capacity to see new patients.^54^ In that study, patients deemed “non-urgent” and who had waited the longest for local geriatric services were referred to the ICT for assessment and risk stratification. Of 138 patients, 27 were re-triaged as urgent and promptly seen by the ICT team geriatrician, who only felt the need to see one of these in follow-up, whereas usual practice is for almost all patients to receive at least one follow-up visit.^54^ Second, the ICT creates a primary care nexus for a network of health and social care providers, more efficiently leveraging existing community expertise and supports. Finally, the ICT builds self-care capacity among patients and care partners, essential to prevent ED visits.

By specifically targeting older adults with complex needs, and meeting these needs with commensurate clinical resources, the ICT applies the principles of chronic disease management and prevention.^7^ This approach is analogous to that of the Geriatric Resources for Assessment and Care of Elders Primary Care Model for low-income seniors in Indiana and which has sustained reductions in acute care utilization of a similar magnitude to those realized by the ICT.^55^ Rates of ED visits among patients in the ICT, as shown in our study, compare favourably to those found by Manis et al. for community-dwelling older persons and older home care clients in 2019 (∼375 visits per 1,000 population and ∼830 visits per 1,000 patients), respectively.^56^ Other work has shown how embedding specialists within primary care teams, including geriatricians, can contribute to better patient outcomes.^56-58^ The ICT represents a useful template for building system capacity to better support patients with other complex conditions, such as chronic pulmonary conditions, heart failure, and mental health disorders.

This work has limitations. First, it describes findings from a single primary care site and model. Future work is needed to understand how to apply these findings to other primary care settings with different resources. Moreover, additional work is required to better understand the impact of the ICT on patient health-related quality of life and caregiver burden. However, our findings underline the importance of interprofessional capacity in primary care to better support older adults with complex needs. Second, embedding geriatricians in primary care is a departure from usual practice and may require modification of existing approaches to remuneration. Third, ensuring the spread and sustainability of the ICT requires a quality assurance framework. The Check-Up may lend itself to the development of quality indicators, as was done with other interRAI instruments.^59-62^ Finally, the ICT does not support older adults with lower AUA scores but at risk of becoming frailer, and for whom targeted preventative services need to be defined. Further work is needed to configure primary, specialist, and community care to support this population.^63^

## Conclusion

The ICT, a primary care interprofessional shared-care program, can improve outcomes for community-dwelling older adults with complex needs. The ICT succeeds through integrated interprofessional care processes that support patient and care partner self-care skills.

## Data Availability

Due to the small sample size, we prefer to discuss data sharing on a case-by-case basis. Please contact the corresponding author.*
